# Immune correlates of therapy outcomes in women with cervical cancer treated with chemoradiotherapy: A systematic review

**DOI:** 10.1002/cam4.4017

**Published:** 2021-06-12

**Authors:** David S. Lakomy, Juliana Wu, Dorothy Lombe, Emmanouil Papasavvas, Susan Citonje Msadabwe, Yimin Geng, Luis J. Montaner, Elizabeth Chiao, Lilie L. Lin

**Affiliations:** ^1^ Departments of Radiation Oncology The University of Texas MD Anderson Cancer Center Houston TX USA; ^2^ Dartmouth Geisel School of Medicine Hanover NH USA; ^3^ The University of Texas School of Public Health Houston TX USA; ^4^ Cancer Diseases Hospital Lusaka Zambia; ^5^ Departments of Immunology, Microenvironment & Metastasis Program The Wistar Institute Cancer Center Philadelphia PA USA; ^6^ Research Medical Library The University of Texas MD Anderson Cancer Center Houston TX USA; ^7^ Departments of General Oncology The University of Texas MD Anderson Cancer Center Houston TX USA; ^8^ Departments of Epidemiology The University of Texas MD Anderson Cancer Center Houston TX USA

**Keywords:** immunologic factors, biomarkers, cervical cancer, HIV, immune exhaustion, lymphocytes, prognosis

## Abstract

**Background:**

Immune markers have been correlated with prognosis in a variety of solid tumors, including cervical cancer.

**Objective:**

To review the literature on hematologic and immune markers and their association with recurrence and survival among patients with cervical cancer treated with chemoradiation.

**Evidence review:**

This systematic review was conducted in accordance with PRISMA guidelines via searches of Ovid MEDLINE, Ovid Embase, and the Cochrane Library using keywords regarding cervical cancer, immune markers, and HIV. Studies involving patients treated with cisplatin‐based chemoradiotherapy were selected and reviewed by at least two independent reviewers, with disagreements resolved by a third reviewer.

**Findings:**

A total of 737 studies were identified, of which 314 assessed immune biomarkers in immunocompetent patients (30 included in the final analysis) and 327 studies in immunosuppressed patients (5 included in the final analysis). The strongest prognostic indicators were lymphopenia and elevated neutrophil‐to‐lymphocyte ratio. Other potential markers included HPV‐specific lymphocyte response, cytokine profile, expression of immune‐blocking antigens on cell surfaces, and tumor‐associated lymphocyte, macrophage, and neutrophil infiltration. Studies of immunosuppressed patients described more severe cytopenic changes overall and concluded that viral suppression led to improved outcomes.

**Conclusions:**

The immunologic interplay at work in cervical cancer development, progression, and treatment is complex. Strong evidence was found in favor of lymphopenia and elevated neutrophil‐to‐lymphocyte ratio being prognostic for worse outcomes with other markers showing potential associations as well. Although the interpretation of immune status with regard to treatment approach remains unclear, future studies should aim to tailor treatment that minimizes possible detrimental immune effects.

## Abstract 2

Immune markers have been correlated with prognosis in a variety of solid tumors, including cervical cancer. In this systematic review of immune markers for cervical cancer patients being treated with chemoradiation, we surveyed the literature for immunologic and hematologic prognostic markers and found the strongest negative prognostic indicators were lymphopenia and elevated neutrophil‐to‐lymphocyte ratio.

## 1 INTRODUCTION

Understanding of the importance of the immune system in cancer development, progression, and therapeutic response has increased greatly over the past decade. In particular, tumor‐specific immune responses, as well as non‐specific systemic inflammatory responses, have been correlated with treatment‐related outcomes in a variety of solid tumors,^1^ including cervical cancer.^2^, ^3^ These observations underscore the importance of immune surveillance throughout the course of cervical cancer care as well as the need for more detailed understanding of the specific factors involved.

The role of the immune system is particularly relevant in the pathogenesis and treatment of cervical cancer because most cases are associated with human papillomavirus (HPV) infection.^4^ In addition, chemoradiation (CRT), which is the standard of care for locally advanced cervical cancer,^5^, ^6^ can result in significant lymphopenia due to the exquisite radiosensitivity of lymphocytes. In geographic regions where the cervical cancer burden is greatest, e.g., in sub‐Saharan Africa,^7^ co‐infection with HIV has been associated with inferior clinical outcomes after CRT,^8^, ^9^, ^10^, ^11^, ^12^, ^13^, ^14^ further underscoring the importance of an intact immune system for positive therapeutic outcomes. Finally, immunotherapies aimed at circumventing intratumoral immune blockade have shown promise in the management of several types of solid malignancies, including cervical cancer.^15^, ^16^

Given the importance of immunologic factors in cancer surveillance, this review examines the role of immune markers in predicting clinical outcomes among patients with cervical cancer receiving CRT. Additionally, we performed a separate review of hematologic and immunologic markers in patients who are living with HIV (PLWH) and receiving CRT for cervical cancer.

## 2 DATA SOURCES AND SELECTION

**FIGURE 1:**
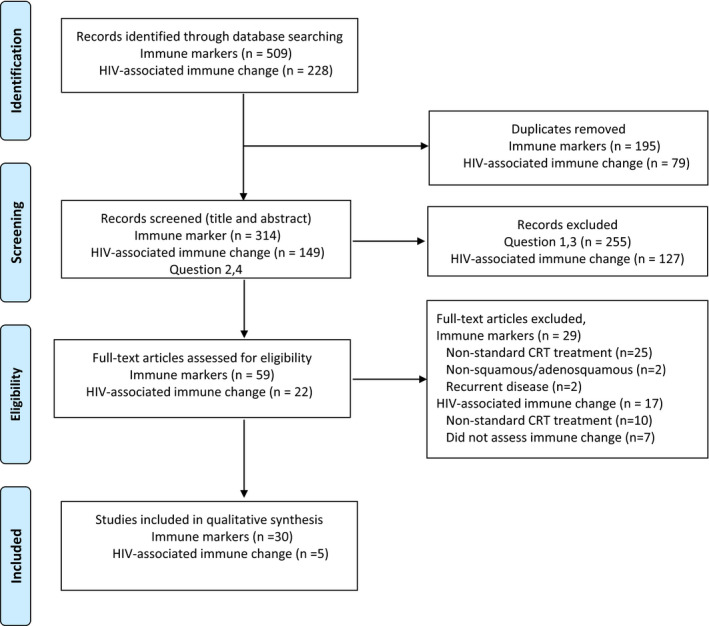
PRISMA diagram

To review immune markers associated with cervical cancer, we undertook a systematic review in accordance with the Preferred Reporting Items for Systematic Reviews and Meta‐Analyses (PRISMA) guidelines.^17^ An electronic search of the following databases was performed for each research question namely, relationships between immune markers and outcome among (a) patients with cervical cancer treated with CRT and (b) patients with cervical cancer and HIV co‐infection treated with CRT: Ovid MEDLINE, Ovid Embase, and Cochrane Library for publications in English from January 2000 to date. The search was first performed in February 2020 and updated in May 2020. To assess the relationship of immune markers and clinical outcomes, the following concepts were searched by using the following subject headings and keywords as needed: “uterine cervical cancer,” “radiotherapy”, “survival analysis”, “survival rate”, “adverse events”, “lymphocytes”, “lymphocyte count”, “lymphocyte ratio, “lymphopenia”, “neutrophils”, “leukocytes”, “platelet count”, “T‐lymphocyte differentiation antigens”, “immunologic cytotoxicity”, “cellular immunity, “immune”, “immunity” and “biomarkers”. Inclusion criteria required receipt of concurrent platinum‐based CRT in at least 50% of patients; exclusion criteria were having small‐cell or other non‐squamous/non‐adenomatous histology or an exclusive focus on recurrent disease (Figure 1).

To assess immune marker changes in PLWH with cervical cancer who received CRT, the following concepts were added to the search strategy: “human immuno deficiency virus”, “HIV”, “HIV Infections”, “acquired immuno deficiency syndrome”, and “AIDS.” Inclusion and exclusion criteria were the same as those noted above, with documented HIV infection and measurements of hematologic/immune variables throughout treatment. The strategy and full list of terms for an Ovid MEDLINE search are provided in Appendix I.

After removal of duplicates, each study was initially identified by title and abstract review followed by full‐text evaluation. At least two reviewers independently reviewed full articles; the methodological quality of each publication was graded per the NIH National Heart, Lung, and Blood Institute (NHLBI) quality assessment tool for observational cohort studies, and disagreements were resolved by a third reviewer.

A total of 737 studies were identified; removal of 274 duplicates resulted in 463 studies (314 on immune biomarkers and 149 on immune or hematologic changes over cancer treatment in the context of HIV). Subsequent screening of the titles and abstracts for eligibility led to selection of 59 immune‐biomarker studies and 22 HIV‐associated immune/hematologic changes. Of these studies, full‐text review of the 59 immune‐marker studies led to exclusions for non‐standard CRT (n = 25) or for having non‐squamous / adenomatous disease (n = 2) or recurrent disease (n = 2). Of the 22 studies of immune changes in HIV‐positive patients, the most common exclusions were non‐standard CRT (n = 10) or not having reported changes in hematologic or immune variables (n = 7). The final numbers of studies included in the qualitative synthesis were 30 on immune markers and 5 on immune/hematologic markers associated with HIV.

Among the 30 immune‐marker studies, most focused primarily on standard hematologic venous markers (n = 22) vs non‐standard markers (n = 9), with one study including both. The immune markers studied most often were lymphocytes (n = 15) or neutrophil‐to‐lymphocyte (NLR) ratios (n = 9). In the 5 studies of immune changes in immunosuppressed (i.e., HIV‐infected) patients, assessed total leukocyte count was reported in 3, absolute neutrophil count (ANC) in 2, and ALC/CD4 count in 2.

A summary of the quality assurance grading system is reported in Appendix II. Overall, most studies (71%) were retrospective. Areas of bias included selection, with studies focusing on a narrow selection of cervical cancer stages (43%), and reliance on single exposure measures rather than repeat or longitudinal measures (63%).

## 3 FINDINGS AND DISCUSSION

The immune system has a central role in cancer development, including the targeting and elimination of neoantigens. Local and systemic inflammatory processes are known to intersect with a variety of tumor‐promoting effectors including growth factors, proangiogenic factors, extracellular matrix‐modifying enzymes, and mutagenic molecules that all have individual and overlapping roles with tumor formation, progression, invasion, and metastasis.^1^, ^18^ Although there are innumerable immune markers to examine, we focused on two general types: hematologic markers that are easily obtained via a standard‐of‐care peripheral‐blood differential analysis (Table 1) and other markers requiring more sophisticated techniques to assess, such as immunohistochemical staining, flow cytometry, and other non‐standard‐of‐care assays (Table 2).

**TABLE 1:** Studies of standard hematologic immune markers as prognostic oncologic determinants for patients with cervical cancer treated with definitive platinum‐based chemoradiation

Study and reference	No. of patients	FIGO disease stage	Immune markers assessed	Time points	Multivariate Analysis of Each Marker/Timepoint as an Independent Prognostic Factor
Cho et al (2016)^26^	124	I – III	ALC	Baseline, on‐treatment nadir	ALC <200 cells/μL during treatment prognostic for worse PFS and DSS (HR 3.28, 95% CI 1.27–8.48, *p *= 0.014; HR 3.0, 95% CI 1.37–9.44, *p *= 0.009) ALC ≤1500 cells at baseline not associated with worse PFS or DSS
Mizunuma et al (2015)^42^	56	I‐IV	NLR	Baseline	NLR ≥ 2.5 at baseline associated with worse OS and PFS (HR 2.80, 95% CI 0.838–9.34, *p *= 0.005; HR 1.53, 95% CI 1.19–1.97, *p *= 0.001)
Jonka‐Gmyrek et al (2018)^39^	94	IA‐IV	NLR	Baseline	NLR>1.6 at baseline associated with worse OS and RFS (HR 2.85, 95% CI 2.011–3.685, *p *= 0.014; HR 3.0, 95% CI 2.063–3.936, *p *= 0.021)
Holub & Biete (2019)^44^	151	IA2‐IVB	NLR, PLR, Eosinophil, ELR	Baseline	ELR≥0.07 at baseline associated with improved 5‐yr OS (HR 0.49, 95% CI 0.31–0.98, *p *= 0.048) but not PFS Eosinophils ≥280 cells/μL and NLR ≥3.8 not associated with 5‐yr OS or PFS
Glicksman et al (2017)^35^	287	IB‐IIIB	ANC	Baseline, on treatment	ANC on treatment, treated as a continuous variable, prognostic for DFS (HR 1.17, *p *= 0.042). Baseline ANC, treated as a continuous variable, not associated with DFS
Choi et al (2008)^a^, ^22^	143	IB2‐IVA	ALC, WBC	Baseline	ALC at baseline, as a continuous variable, prognostic for improved PFS and for an association with CR (HR 0.42, 95% CI 0.20–0.89, *p *= 0.023; OR 3.08, 95% CI 1.93–11.35, *p *= 0.010) Risk of progression decreased by 58% for every 1000 cells/μL increase in ALC
Hoskin et al (2014)^20^	111	IB‐IVA	ALC	Baseline	ALC ≥1600 cells/μL at baseline prognostic for improved 5‐yr OS and LRC (HR 0.43, 95% CI 0.243–−0.737, *p *= 0.002; HR 0.463, 95% CI 0.505–0.792, *p* =0.01)
Haraga et al (2016)^82^, ^a^	131	IB1‐IVA	NLR	Baseline	NLR ≥2.85 at baseline not associated with OS or PFS
Koulis et al (2017)^83^	257	IB‐IVA	NLR	Baseline, on treatment (average)	NLR>5 at baseline not associated with 5‐yr OS or 5‐yr PFS
Lee et al (2019)^32^	92	IB‐IVA	RTL (i.e. relative rate of lymphocyte loss over treatment)	On‐treatment	Patients with poor RTL had worse 3‐year PFS (HR 3.28, 95% CI 1.17–9.2, *p *= 0.024) and DSS (HR 5.06, 95% CI 1.25–20.5, *p *= 0.023)
Wisdom et al (2019)^34^	278	IB1‐IVA	ANC	Baseline, on treatment (week 1)	ANC >8000 cells/μL on treatment associated with worsened CSS, cervical recurrence, pelvic recurrence (HR 1.12, 95% CI 1.03–1.19, *p *= 0.007; HR 1.15, 95% CI 1.04–1.27, *p *= 0.006; HR 1.14, 95% CI 1.04–1.25, *p *= 0.005) but not distant recurrence ANC >8000 cells at baseline not associated with CSS, cervical recurrence, pelvis recurrence, or distant recurrence
Taguchi et al (2020)^25^	168	IB1‐IVA	ALC	Baseline, treatment end, 6‐month FU	ALC<600 cells at 6‐month FU associated with worse OS and PFS (HR 3.06, 95% CI 1.48–6.33, *p *= 0.0026; HR 2.67, 95% CI 1.43–4.99, *p *= 0.0021) ALC 1270 cells/μL at baseline not prognostic for OS or PFS ALC<290 cells at treatment end not associated with OS or PFS
Petrillo et al (2015)^61^, ^a^	84	IB2‐IVA	AMC	Baseline	AMC at baseline, as a continuous variable, elevation associated with worse OS and DFS (HR 1.181, *p *= 0.041; HR 1.462, *p *= 0.043)
Wu et al (2016)^24^	47	IB2‐IVA	ALC	Baseline, 2‐month FU	ALC ≥1000 cells/μL at baseline possibly associated with improved OS (*p *= 0.053) but not PFS ALC ≥500 cells/μL at 2‐month FU not prognostic for OS or PFS
Onal et al (2016)^40^	235	IB2‐IVA	NLR	Baseline	NLR ≥3.03 at baseline associated with worse OS and PFS (HR 3.322, 95% CI 1.905–5.790, *p *< 0.001; HR 3.579, 95% CI 2.106–6.082, *p *< 0.001)
Onal et al (2018)^23^	95	IB2‐IVA	ALC	Baseline, 2‐month FU	ALC<1000 cells/μL at baseline associated worse 5‐yr OS (HR 4.59, 95% CI 1.99–10.62, *p *< 0.001) and PFS (HR 5.87, 95% CI 2.44–14.10, *p *< 0.001) ALC<500 cells/μL at 2 months was not associated with worse 5‐yr OS or PFS
Escande et al (2016)^36^	113	IB2‐IVB	ANC	Baseline	ANC>7500 at baseline associated with worse in‐field FFS and local FFS (HR 4.50, *p *= 0.002; HR 3.1, *p *= 0.018) but not OS or distant FFS
Wang et al (2016)^41^	60	II‐III	NLR	Baseline	NLR ≥2.0 at baseline associated with worse OS (HR 0.268 95% CI.0.78–0.924, *p *= 0.037)
Li et al (2016)^21^, ^a^	147	IIA‐IVA	ALC, AMC, LMR	Baseline	ALC ≥2350 cells/μL at baseline prognostic for improved 5‐yr OS and 5‐yr PFS (HR 0.458, 95% CI 0.198–0.824, *p *= 0.021; HR 0.512, 95% CI 0.165–0.928, *p *= 0.013) LMR>5.28 at baseline prognostic for improved 5‐year OS and 5‐yr PFS (HR 0.251, 95% CI 0.161–0.412, *p *= 0.013; HR 0.218, 95% CI 0.014–0.582, *p *= 0.021) AMC ≥380 cells/μL at baseline not significant for 5‐yr OS or 5‐yr PFS
Cho et al (2017)^43^	105	IIB	NLR	On treatment (2^nd^ week)	NLR>3.59 on 2^nd^ week of treatment not associated with 5‐yr DSS or 3‐yr PFS
Lee et al (2020)^37^, ^a^	125	IIB‐IIIB	ALC, AMC, ANC, NLR, MLR,	Baseline, 2–4 weeks after treatment completion)	NLR>5.231 at treatment completion prognostic for worse OS and DFS (HR 12.639, 95% CI 4.969–32.144, *p *< 0.001; HR 3.643, 95% CI 1.701–7.802, *p *= 0.001) Baseline and after‐treatment ALC, ANC, AMC, MLR not prognostic for OS or DFS
Jeong et al (2019)^27^	392	IIB‐IVA	ALC, Lymphocyte %, ANC, Neutrophil %, NLR	Baseline	Lymphocyte %>24 prognostic for improved 5‐yr PFS (HR 0.59, 95% CI 0.40–0.85, *p *= 0.005) but not OS NLR >2.8 prognostic for worse 5‐yr PFS (HR1.55, 95% CI 1.07–2.25, *p *= 0.022) but not OS ALC>1870 cells/μL, ANC ≥5510 cells/μL, Neutrophil % >65% not prognostic for OS or PFS

All studies had at least ≥50% of patients who were treated with platinum‐based chemoradiotherapy.

Abbreviations: absolute monocyte count; AMC, absolute lymphocyte count; ALC; ANC, absolute neutrophil count; CI, confidence interval; CR, complete response; DSS, disease‐specific survival; ELR, eosinophil‐to‐lymphocyte ratio; FFS, failure‐free survival; FU, follow‐up; HR, hazard ratio; LMR, lymphocyte‐to‐monocyte ratio; NLR, neutrophil‐to‐lymphocyte ratio; NS, non‐significant (determined as *p* > 0.05); OS, overall survival; PFS, progression‐free survival; RTL, relative tolerance of lymphocytes; yr, year.

^a^ Denotes studies with additional concurrent chemotherapy regimens (e.g., Choi et al, 2008 cisplatin and flurouracil; Haraga et al, 2016 cisplatin with or without ifosfamide; Petrillo et al, 2015 cisplatin and flurouracil; Li et al, 2016 cisplatin and flurouracil; Lee et al, 2020 cisplatin with vincristine and mitomycin or cisplatin with cyclophosphamide).

**TABLE 2:** Studies of non‐standard‐of‐care immune markers as prognostic oncologic determinants for patients with cervical cancer treated with definitive chemoradiation

Study and reference	No of patients	FIGO disease stage	Immune markers assessed	Central outcomes
Peripheral				
Ordoñez et al (2013)^46^	58	I‐II	Radiation‐induced apoptosis value of peripheral CD8+ cells	↑ Radiation‐induced apoptosis for CD8+ T cells associated with worse DFS and CSS (OR 12.915, 95% CI 2.837–58.794, *p *= 0.001; OR 7.805, 95% CI 1.243–48.991, *p *= 0.028)
Yang et al (2006)^52^	42	IBI‐III	Baseline serum levels of TGF‐β1, VEGF, IL−6	Baseline TGF‐β1<30 ng/mL and VEGF <70 pg/mL associated with complete or partial tumor response (OR 3.1, *p *= 0.04; OR 2.7, *p *= 0.05) IL−6 >35 pg/mL not associated with response
Delgado et al (2009)^51^	32 (16 controls)	IBI‐IIIB	IFN‐γ and IL−4 cytokine response from lymphocytes stimulated with HPV−16 E7 peptides at baseline and after treatment	↓Th2 response at baseline associated with significant decrease in DFS (*p *= 0.027) Not observed with Th2 response after treatment or with Th1 responses at any time
Ma et al (2018)^45^, ^a^	66 (60 controls)	IIB‐IIIB	HPV E1‐specific T‐cell response (PBMCs were isolated and stimulated with whole E1‐protein and measured with an IFN‐γ ELISA)	↑HPV E1‐specific T‐cell response an independent prognostic factor for improved OS and PFS (HR 7.499, 95% CI 1.661–33.856, *p *= 0.009; HR 7.252, 95% CI 1.690–31.126, *p *= 0.008)
Tumor‐associated				
Tsuchiya et al (2020)^56^	104	I‐IV	PDL1‐expressing tumor cells Tumor‐associated CD8+, FoxP3+ cells	↑ PDL1 expression on tumor cells after treatment associated with improved OS (HR 0.224, 95% CI 0.096–0.525, *p *< 0.001) and reduced out‐of‐field recurrence (*p *= 0.005) ↑ CD8+ and FoxP3+ T‐cell infiltration within the tumor microenvironment at baseline associated with better OS on univariate analysis (HR 0.260, 95% CI 0.108–0.628, *p *= 0.003, HR 0.286, 95% CI 0.120–0.683, respectively)
Matsumoto et al (2017)^64^	250	IA2‐IVB	Tumor‐associated neutrophils (CD66b+ cells)	↑ Intratumoral neutrophil density associated with worse PFS (HR 4.95, 95% CI 2.51–10.7, *p *< 0.0001)
Cosper et al (2020)^60^	115	IB1‐IIIB	Before‐treatment and mid‐treatment analysis of: PD1, PDL1, PDL2, CTLA4 expression, and genetic expression differences as a response to treatment CD8+ cells within the microenvironment T‐cell expression of: CD3e, CD4, CD8a, PRF1, GAMA, GNLY	655 genetic changes vs 5533 after treatment in those who DoD vs had NED; 67% and 80% of immune‐related pathways enriched at before treatment and mid‐treatment vs 0% and 0.6% at before treatment and mid treatment for patients NED vs DOD (*p *< 0.01) ↑CTLA4 and PD1 expression in patients with NED vs those who DoD who had increased expression of PDL1 and PDL2 at mid‐treatment ↓Intratumoral lymphocytes in patients who DoD vs those who had NED (2% vs 9%, *p *= 0.01) ↓ Expression of CD3e, CD4, CD8a, PRF1, GAMA, GNLY (markers of cytolytic activity) in patients who DoD vs those who had NED
Petrillo et al (2015)^61^, ^a^	84	IB2‐IVA	Tumor‐associated macrophages (M1 and M2 phenotypes)	↑ M1/M2 associated with improved 5‐yr OS (69.3% vs 46.9%; *p *= 0.037) and 5‐yr DFS (67.2% vs. 44.3%; *p *= 0.019) vs ↓ M1/ M2 Total TAM count not associated with oncologic outcome
Martins et al (2019)^57^	21	IIB‐IIIB	Tumor associated CD8+ T cells Tumor‐associated macrophages (CD68+ cells) PD1, PDL1, and PDL2 expression on TILs	↑ CD8+ T cells in responders vs non‐responders in both the stroma and peri/intratumoral region (*p *< 0.05) Macrophage infiltration no different in the stroma and peri‐intratumoral region between responders vs non‐responders ↑ PDL1+ expression on TILs in responders (*p *< 0.01); responders had ↑PDL1+ TILs within the stroma vs the peri/intratumoral region vs non‐responders (*p *< 0.01)

All studies had at least ≥50% of patients who were treated with platinum‐based chemoradiotherapy.

Abbreviations: CI, confidence interval; CSS, cancer specific survival; DFS, disease‐free survival; DoD, died of disease; ELISA, enzyme‐linked immunosorbent assay; FoxP3+, Forkhead box P3; GAMA, Ganzyme A; GNLY, Granulysin; HPV, human papillomavirus; HR, hazard ratio; IFN‐γ, interferon‐γ; IL‐, interleukin; NED, no evidence of disease; OR, odds ratio; PBMC, peripheral blood mononuclear cell; PDL, programed cell death ligand; PFS, progression‐free survival; PRF1, perforin 1; TAM, tumor‐associated macrophage; TGF‐, tumor growth factor‐; Th‐, t‐helper cell; TILs, tumor infiltrating lymphocytes; VEGF, vascular endothelial growth factor.

^a^ Denotes studies with additional concurrent chemotherapy regimens (Petriollo et al, 2015, cisplatin and flurouracil; Ma et al 2018, cisplatin with or without paclitaxel).

### 3.1 Standard‐of‐care hematologic immune markers

Studies assessing standard hematologic immune markers are particularly plentiful as these markers are relatively inexpensive to obtain, are accessible, and can be measured repeatedly throughout therapy and follow‐up. Although the results studied do not yield a complete picture of the immune system, they nevertheless provide a basic overview that is easily clinically translated to clinical practice.

#### 3.1.1 Lymphocytes

The concept of immunosurveillance wherein immune cells, primarily lymphocytes, can identify and eliminate nascent malignant cells was proposed as early as the 1950s.^19^ Two central questions regarding the prognostic value of lymphocyte count are what thresholds and what time points throughout treatment are most informative in predicting outcomes. Some investigators have found that low absolute lymphocyte counts (ALC) at baseline (i.e., before treatment is begun) ranging from 1000 to 2350 cells/μL are associated with poor outcomes, including attenuated overall survival (OS), progression‐free survival (PFS), and worse locoregional control.^20^, ^21^, ^22^, ^23^ However, other studies using similar thresholds (1000–1870 cells/μL) did not show comparable results.^24^, ^25^, ^26^, ^27^

Having low baseline lymphocyte counts suggests the presence of aberrant systemic T‐cell homeostasis influenced by each individual's cancer‐derived microenvironment and may represent a fixed characteristic for each patient.^28^ However, changes in lymphocyte counts during treatment and at recovery in patients with cervical cancer treated with standard‐of‐care definitive CRT can be strongly influenced by the treatment itself. Lymphocytes are among the most radiosensitive cells in the body,^29^ and lymphopenia can result from irradiation of circulating lymphocytes^30^ and bone marrow.^31^ Research regarding lymphopenia nadir values during treatment is less robust than studies evaluating baseline lymphocyte count, with conflicting results in the literature.^25^, ^26^ That said, one group reported that the rate of lymphocyte count change throughout treatment, specifically a more rapid relative loss of lymphocytes from baseline, can itself be prognostic of PFS and disease‐specific death.^32^

Changes in ALC after treatment, relative to baseline, should also be considered, as recovery of circulating lymphocytes may have prognostic influence. One study showed that ALC levels of ≥500 cells/μL at 2 months after therapy may be associated with improved OS and PFS,^24^ and another found that ALC <600 cells/μL at that time was associated with inferior OS and PFS.^25^ Thus, although lymphocyte count has been shown to be prognostic, the precise timepoints and thresholds are still unclear. The gist seems to be that higher lymphocyte counts before, during, and after recovery from therapy are associated with better prognosis.

#### 3.1.2 Neutrophils

Unlike lymphocytes, which serve to eliminate neoantigens, neutrophils have multifaceted roles in tumor initiation, growth, and metastasis,^33^ and some evidence exists to suggest that neutrophils may promote radiation resistance.^34^ Indeed, higher neutrophil counts at baseline generally portend worse prognosis.^33^ Similar to lymphocytes, the levels and time points at which absolute neutrophil count (ANC) are most predictive remain unclear. Findings from studies of baseline neutrophilia are mixed,^27^, ^34^, ^35^, ^36^, ^37^ although some studies have found that elevated ANC during treatment was associated with worse DFS^35^ and with lower rates of local control, metastasis‐free survival, and cause‐specific survival.^34^ Thus although the predictive power of ANC levels at baseline remains debated, rises in ANC during therapy likely indicate worse outcomes.

More than ALC or ANC alone, the combination of neutrophilia and relative lymphopenia—often reported as the neutrophil‐to‐lymphocyte ratio (NLR)—has been more robustly associated with poor outcomes in gynecologic malignancies.^3^ NLR is thought to be a proxy for the systemic balance between the pro‐inflammatory effects of neutrophils and the antitumor immune response of lymphocytes, and thus can describe simultaneous trends in greater tumorigenic activity and decreased antitumor capacity.^38^ Several groups have evaluated NLR at baseline and generally found significantly worse outcomes associated with NLR values from 1.6 to 3.03.^27^, ^39^, ^40^, ^41^, ^42^ Post‐treatment NLR (or the change in NLR from baseline) has not been assessed as rigorously, and findings are more ambiguous.^37^, ^43^ In sum, NLR values at baseline may be informative, but how changes during and after therapy can better inform outcomes requires additional study.

#### 3.1.3 Eosinophils and monocytes

Eosinophils and monocytes have also been assessed as markers of inflammation akin to neutrophils. Thus far little prognostic utility has been found for either cell type measured individually; however, in combination with lymphocytes (i.e., eosinophil‐to‐lymphocyte [ELR] or lymphocyte‐to‐monocyte [LMR] ratios), they have shown some association with outcomes. For example, a baseline ELR of >0.07 was correlated in one study with improved 5‐year OS,^44^ and a LMR >5.28 was shown in another to predict improved 5‐year OS and PFS.^21^

### 3.2 Non‐standard‐of‐care peripheral and intratumoral immune markers

Beyond the information to be gained from a typical venous blood differential analysis, the ability to detect and identify not only specific immune cells but also qualitative changes in gene expression and cell‐associated protein expression associated with immune regulation has enhanced the understanding of immune dynamics in cervical cancer, as described below.

#### 3.2.1 Peripheral markers

##### Lymphocyte response

Functional assays have suggested some association between peripheral lymphocytes and response to cervical cancer therapy. In one study, higher activity of T cells (extracted before treatment) in response to HPV E1‐specific antigens was linked with improved OS and PFS.^45^ Another study examined the radiosensitivity of CD8+ T cells extracted before treatment and found that patients whose T cells had higher rates of radiation‐induced apoptosis (i.e., higher lymphocyte radiosensitivity) had worse disease‐free and cancer‐specific survival.^46^ Interestingly, the same study found no links between other lymphoid immune cells including CD4+ T cells, B cells, and natural killer cells and prognosis.^46^ Collectively, these findings support the importance of the CD8 T‐cell subset, with its emphasis on antigen‐specific responses, as a positive indicator of cancer‐free survival.

##### Cytokine profiles

Although immune cells ultimately execute immune functions (e.g., antigen presentation, surveillance, cytotoxicity), it is cytokines, a broad group of soluble proteins, that orchestrate immune cell migration patterns, polarization/differentiation, and overall activity. The cytokine profile produced by CD4+ T‐helper cells in particular, i.e., the Th1/Th2 cytokine balance, is important in the prognosis of several types of cancer.^47^, ^48^, ^49^, ^50^ Delgado et al. found that cervical cancer patients whose peripheral lymphocytes expressed a Th2‐type response after HPV‐specific antigen stimulation before treatment had significantly worse DFS (*p *= 0.027) and perhaps worse OS as well (*p *= 0.126) at 40 months.^51^ Overall, a higher Th1/Th2 ratio has been linked with better outcomes in cervical cancer. This relationship between Th1 cytokine profiles and better prognosis is also consistent with findings from other studies of type 1 tumor‐associated macrophages (TAMs) and profiles favoring cytotoxic CD8 T‐cell infiltration (i.e., Th1‐induced), as reviewed in the tumor microenvironment markers section below.

Studies of the angiogenic factors tumor growth factor‐β (TGF‐β1) and vascular endothelial growth factor (VEGF) have also shown that having lower levels of both before CRT for cervical cancer has been associated with complete or partial tumor response.^52^ Possible mechanisms for this observation are that attenuated TGF‐β1 levels result in decreased cell proliferation^53^ and restriction of T‐cell infiltration,^54^ whereas decreased VEGF levels restrict endothelial cell proliferation and permeability.^55^

#### 3.2.2 Tumor microenvironment markers

### 3.3 Lymphocytes

In addition to functional assays of peripheral lymphocytes, the composition of intratumoral lymphocyte subpopulations (cytotoxic and regulatory T‐cell) has also been assessed in cervical tumor specimen's pre‐treatment for prognostic significance. Infiltration of CD8+ T cells into both the tumor stroma and the peri‐tumoral or intratumoral region has been associated with response and survival.^56^, ^57^ Also, intratumoral infiltration of Forkhead box P3 (FoxP3)+ T cells (that is, regulatory T cells associated with reduced immune activation and enhanced tumor immune escape) evident before treatment has been associated with better OS.^56^ Mechanistically, these improved outcomes may be attributable to co‐association with increased HPV‐16 positivity, co‐infiltration with effector T cells, and increased anti‐inflammatory effects, all known to limit tumor progression.^58^, ^59^ Studies assessing tumor‐associated lymphocyte subpopulations during and after treatment are limited. Cosper et al. found that patients who died of disease after CRT had significantly fewer intratumoral lymphocytes (3% vs 9%, *p *= 0.01) and lower expression of cytolytic lymphocyte genes at mid‐treatment compared with patients with no evidence of disease after treatment.^60^ Establishing direct links among a baseline immunoregulatory‐cell set point in the tumor microenvironment, lymphocyte infiltration, and response to therapy is an ongoing area of investigation.

#### Tumor‐associated macrophages and neutrophils

Although to date no differences have been found in infiltration of myeloid cells within the tumor microenvironment at baseline or after treatment between patients who respond to treatment and those who do not,^57^, ^61^ closer assessment of myeloid polarization patterns has raised the hypothesis that macrophage polarization phenotypes may be prognostic.^61^ Type 1 (M1) TAMs promote inflammatory immune activity by increasing antigen‐presentation capacity and inducing Th1 immunity;^62^ type 2 (M2) TAMs are activated through a variety of pathways, including anti‐inflammatory molecules such as interleukins (IL) −4, IL‐13, and IL‐10.^63^ Consistent with the link between CD8 T‐cell cytotoxic phenotypes and tumor control, greater polarization towards the M1 phenotype (an M1/M2 ≥ 0.600) has been shown to independently predict improved 5‐year OS and DFS.^61^

With regard to neutrophil infiltrates at baseline, increased density of CD66b+ neutrophils within the tumor microenvironment has been linked with shorter PFS.^64^ Future studies are needed to address whether the presence of greater neutrophil‐based inflammation is counter to steady‐state levels of FOXP3 T cells or to the emergence of M1 and CD8 T‐cell infiltrates. For example, whether activated neutrophils act on myeloid suppressor cells within the tumor microenvironment in a way that leads to a poor prognosis remains to be determined.^65^

#### Immune blockade

Cell surface markers associated with downregulation of immune activity, particularly those markers mediated by T‐cell lymphocytes (e.g., programmed cell death protein/ligand‐1/2 [PD/L1/2]) and cytotoxic T‐lymphocyte‐associated protein [CTLA4]), are often upregulated on tumor cells.^15^, ^16^ The effects of blocking the cell‐surface expression of immune markers have been assessed both before and after CRT for cervical cancer, with studies showing mixed to negative findings. In one study, patients who responded to standard CRT had an overall increase in PDL1, but not in PD1 or PDL2 expression, on tumor‐infiltrating lymphocytes (TILs) at baseline relative to patients who did not respond.^57^ A separate study also found that decreased PD1 expression at baseline may have been associated with worse OS (*p *= 0.116).^56^ Analysis of RNA expression patterns during CRT showed a mixed picture in which patients who died of disease had increase expression of PDL2 but decreased expression of CTLA4 and PD1.^60^ Finally, others have found that increased PDL1 expression on tumor cells after CRT was associated with improved OS.^56^ The heterogeneity of these results suggests that enhanced expression of immune blockade markers could be correlated with an active immunoregulatory environment, perhaps dependent on PD1 interactions, a concept that would support the efficacy of anti‐PD1 as immunotherapy.

### 3.4 Role of HIV

The global burden of cervical cancer is increasingly shifting to locations where HIV co‐infection is more common.^7^ Treatment of locally advanced cervical cancer does not vary based on HIV status—or, indeed, on receipt of anti‐retroviral therapy (ART). Nevertheless, due to the inherent immunologic impacts caused by HIV, this raised the question of how HIV may alter the various links between immune markers and prognostic outcomes detailed above. While no studies in our search directly answered this question, there have been separate analyses of women living with HIV (WLHW) who have received chemoradiotherapy for cervical cancer in which acute immune response to treatment is assessed.

#### 3.4.1 Immune response to chemoradiation for women living with HIV with cervical cancer

Studies assessing changes in immune markers among WLWH are fairly sparse, with most having limited sample sizes (Table 3); nevertheless, cytopenic changes seem to be more severe among WLWH than in women who are HIV‐negative.^66^, ^67^ One report of 29 WLWH (90% of whom were on ART) showed that although ALC (and thus absolute CD4 cell count) was reduced in conjunction with an absolute reduction in total WBC, the percentage of CD4+ cells (CD4%) was not affected by radiation.^68^ These results suggest that CRT itself should not specifically affect immune function in HIV disease. However, intensified neutropenia has also been observed in WLWH in several separate studies.^67^, ^69^

**TABLE 3:** Immune changes in women living with HIV undergoing chemoradiation for cervical cancer

Study and reference	No of patients, total (WLWH)	FIGO disease stage	% of WLWH receiving ART	Immune markers assessed	Central outcomes
Siraprapasiri et al (2011)^68^	29 (29)	NA	96%	WBC, CD4, CD4%, ALC%, HIV‐VL	No change in CD4% from baseline to last week of RT ↓ WBC (6771 vs 3903 cells/μL), ALC% (31.7% vs 17.5%), CD4 (388 vs 158 cells/μL) from baseline to last week of RT Four patients (14%) had increased HIV‐VL after treatment (2 on ART, 2 not on ART)
Grover et al (2018)^67^	143 (96)	I‐IV	95.8%	WBC, ANC	No difference in WBC and ANC nadir during treatment between WLWH and HIV‐negative women
Vendrell et al (2018)^69^	61 (6)	IB‐IV	67%	ANC	Possibly increased risk of grade 3–4 neutropenia during treatment for WLWH vs HIV‐negative women (16.7% vs 3.6%, *p *= 0.27)
Simonds et al (2015)^66^	213 (36)	IB1‐IIIB	100%	WBC, ANC	↑ in rates of grade 3–4 leukopenia (30.6% vs 10.2%, *p *= 0.003) for WLWH vs HIV‐negative women
Einstein et al (2019)^70^	40 (40)	IB2‐IVA	100%	ALC	All patients had decreases in lymphocyte counts that were largely self‐limiting and manageable

Abbreviations: ALC, absolute lymphocyte count; ANC, absolute neutrophil count; ART, antiretroviral therapy; HIV‐VL, HIV viral load; RT, radiation therapy; WBC, white blood cell; WLWH, women living with HIV.

Importantly, most of the WLWH in the published studies were immunologically stable on ART with suppressed viremia,^67^, ^69^, ^70^ although one group found that four patients (14%) had an increased HIV viral load after CRT, with two patients on ART and two not on ART.^68^ Generally, viral suppression while taking ART portends successful completion of therapy; for example, the AIDS‐Malignancy‐Consortium‐081 study found that 82% of WLWH were able to complete anti‐cancer therapy.^70^ However, the effects of residual immune activation for patients taking ART in response to therapy remain to be investigated, as cancer, HIV coinfection, and HPV coinfection have all been reported to increase activation despite ART.^71^, ^72^

#### 3.4.2 Immune exhaustion and HIV

One potential explanation for why WLWH, even with viral suppression while on ART, often have worse outcomes after therapy for cervical cancer despite having minor changes in immune cell populations is immune exhaustion. The idea that dysregulation of T cells can prompt a state of “exhaustion” that limits response to therapy has been of growing interest. Immune exhaustion results from the progressive loss of effector T cells in the presence of continued antigen exposure. First described in chronic infections such as HIV,^73^, ^74^ this process has become increasingly well‐documented in cancer as well.^75^ Under normal circumstances, naïve T cells, when activated through appropriate antigen presentation, are transformed into effector T cells, which have cytotoxic activity.^76^, ^77^ Once antigen clearance is achieved and inflammation is resolved, most T cells are then eliminated, leaving behind a small pool of memory T cells.^76^, ^77^ However, persistent antigen presentation and inflammation (as is seen in HIV and cancer) can result in an alternative end state, that is, T‐cell exhaustion.^76^, ^77^ Although exhausted T cells are not “inert”, in that some residual function remains present, exhaustion is characterized by expression of inhibitory cell surface molecules and a general inability to eradicate pathogens or to mitigate tumor progression.^77^

## 4 CONCLUSION AND FUTURE DIRECTIONS

The immunologic interplay in cervical cancer development, progression, and treatment response is inherently complex. The numerous factors identified to date as having prognostic influence (Table 4) rarely have singular functions and often interact in intricate synergistic networks. In this systematic review, we outlined the immune markers that have been associated with inferior recurrence and survival after CRT for locally advanced cervical cancer, reviewing both standard hematologic markers and non‐standard markers in peripheral blood and in tumor tissues.

**TABLE 4:** Immune signatures of cervical cancer related to prognosis

	Favorable prognosis	Unfavorable prognosis
Hematological Markers	↑ ALC^20^, ^21^, ^22^ ↑ relative lymphocyte count^27^ ↑ ELR^44^ ↑LMR^21^	↑ ANC^34^, ^35^, ^36^ ↑ relative rate of on treatment ALC decline^32^ ↑ NLR^27^, ^37^, ^39^, ^40^, ^41^, ^42^ ↑ AMC^61^
Other Immune Markers	↑CD8+ T cells in the tumor microenvironment^56^, ^57^ ↑ FoxP3 in the tumor microenvironment^56^ HPV E1‐specific T‐cell response^45^ ↑ tumor‐associated neutrophils (CD66+)^64^ M1>M2 TAMs^61^ Th1>Th2 cytokine balance^51^, ^84^ ↓TGF‐β1^52^ ↓ VEGF^52^	↓ CD8+ T cells^60^ ↓ PRF1, GAMA, GNLY expression by T cells^60^ ↑ lymphocyte radiosensitivity^46^

Abbreviations: ALC, absolute lymphocyte count; AMC, absolute monocyte count; ANC, absolute neutrophil count; ELR, eosinophil‐to‐lymphocyte ratio; FoxP3+, Forkhead box P3; GAMA, granzyme A; GNLY, granulysin; HPV, human papillomavirus; LMR, lymphocyte‐to‐monocyte ratio; NLR, neutrophil‐to‐lymphocyte ratio; PDL, programed cell death ligand; PRF1, perforin; TGF‐, tumor growth factor‐; Th, T‐helper cell; VEGF, vascular endothelial growth factor.

This review has several limitations. Most of the studies evaluating standard hematologic marker panels were retrospective and only utilized a single timepoint. As immune markers may vary greatly over the course of treatment, a single time‐point may not be singularly reflective of a patient's immunological state. Additionally, studies that examined more specific immune markers tended to have small numbers of patients which may limit generalizability. Furthermore, selected studies were limited to patients undergoing CRT; consequently, other adjunctive treatments which may impact survival outcomes were not specifically assessed. Image guided brachytherapy, for example, has shown increased control and survival.^78^ Additionally, we limited the selection of studies to focus on squamous, adenosquamous, or adenocarcinomas. Not only do these histologic variants have diverging prognoses, with adenocarcinomas often portending poorer outcome than squamous carcinomas,^79^ but the exclusion of other histologic variants such as neuroendocrine derived tumors might limit external generalizability of the findings presented. Lastly, it is important to note that while the majority of these studies occurred in academic centers, the global burden of cervical cancer is increasingly in low and middle income countries^7^ where treatment availability may differ from the ones utilized in these studies.

Nevertheless, given the limitations of current staging practices and the auspicious emergence of immune‐modulating therapies, a more nuanced understanding of these immune factors may be of benefit. A deeper understanding of each patient's immune phenotype will be vital in optimizing care for patients, whether that translates into choosing radiation modalities for their ability to minimize immune cell toxicity^80^ or identifying novel systemic therapies that influence the cellular immune milieu in ways that enhance antitumor immune activity.^81^ The goal in any approach to tailoring treatment is to maximize effectiveness while minimizing possible detrimental immune effects by optimizing individual patients’ immune phenotypes, with the ultimate result of enhancing oncologic outcomes for cervical cancer. Given the potential for immune exhaustion among WLWH who are undergoing therapy for cervical cancer, a better understanding of their baseline immune status and the effects of CRT on immune status and thus on recurrence and survival may help to inform treatment for all women with cervical cancer.

## CONFLICTS OF INTEREST

Dr. Lin reports receiving funding from AstraZeneca for an investigator‐initiated clinical trial. The authors report no other disclosures or conflicts of interest.

## AUTHOR CONTRIBUTIONS

L.L, L.M., E.C., and S.M. were involved in manuscript conceptualization. L.L., Y.G, and E.C. drove study methodology with Y.G performing the systematic review process. D.S.L., J.W., S.M, D.L., E.C., managed data curation. D.S.L., L.L., D.L., J.W., E.P. performed the draft preparation with review and supervision from L.L, L.M., E.C., and S.M. All authors commented on and reviewed the final manuscript.

## ETHICS STATEMENT

No ethical approval was sought as this project utilized previously published studies in which informed consent was obtained by primary investigators.

## DATA SHARING POLICY

Data sharing not applicable to this article as no datasets were generated or analyzed during the current study.

## Supporting information

Supplementary Material
