# Supplementary material for: Immune correlates of therapy outcomes in women with cervical cancer treated with chemoradiotherapy: A systematic review
Source: Cancer Med. 2021 Jun 12;10(13):4206–20. doi: 10.1002/cam4.4017 (PMC8267128; doi:10.1002/cam4.4017)
Supplement: Supplementary file 1 — Supplementary Material [file CAM4-10-4206-s001.docx]

**Appendix I. Ovid MEDLINE search strategy**

| **#** | **Searches** |
| --- | --- |
| 1 | Uterine Cervical Neoplasms/ |
| 2 | ((cervix or cervical) adj3 (neoplasm* or cancer or carcinoma*)).ti,ab,kf. |
| 3 | 1 or 2 [uterine cervical cancer] |
| 4 | limit 3 to (english language and yr="2000 -Current") |
| 5 | (animals not (humans and animals)).sh. |
| 6 | 4 not 5 |
| 7 | (rat or rats or mouse or mice or xenograft or cells or murine or rodent* or "cell lines").ti. |
| 8 | 6 not 7 |
| 9 | ((child or children or adolescent or pediatric* or paediatric*) not childhood).ti. |
| 10 | 8 not 9 |
| 11 | (esophageal or oesophageal or esophagus or oesophagus or prostate or "head and neck" or anal).ti. |
| 12 | 10 not 11 [remove preclinical study, pediatric, not uterine cervical cancers etc. ] |
| 13 | exp Radiotherapy/ |
| 14 | (radiotherap* or irradiat* or radiat* or chemoradi* or radiochemo* or chemo-radi* or radio-chemo* or RT or "intensity modulated" or IMRT or EBRT or brachytherapy).ti,ab,kf. |
| 15 | ((proton or "heavy ion" or "carbon ion" or particle) adj3 therapy).ti,ab,kf. |
| 16 | exp Radiotherapy Planning, Computer-Assisted/ |
| 17 | exp Radiation Oncology/ |
| 18 | or/13-17 [radiation therapy] |
| 19 | 12 and 18 [Cervical cancer + radiation therapy] |
| 20 | exp Treatment Outcome/ |
| 21 | Survival/ |
| 22 | exp Survival Analysis/ |
| 23 | Survival Rate/ |
| 24 | Kaplan-Meier.ab. |
| 25 | survival.ti,kf. |
| 26 | survival.ab. /freq=2 |
| 27 | exp Neoplasm Recurrence, Local/ |
| 28 | Recurrence*.ti,ab,kf. |
| 29 | exp Neoplasm Metastasis/ |
| 30 | (Metastasis or Metastases).ti,kf. |
| 31 | ("local control" or "regional control").ab. |
| 32 | exp Radiotherapy/ae, co [Adverse Effects, Complications] |
| 33 | Radiation/ae, co [Adverse Effects, Complications] |
| 34 | exp Radiation Dosage/ae [Adverse Effects] |
| 35 | ((radiotherap* or radio-therap* or (radiation adj3 therap*) or chemoradi* or radiochemo* or chemo-radi* or radio-chemo* or irradiat* or brachytherapy) and (adverse* or toxic* or "side effect*" or safety or injur* or abnormal* or induced)).ti,kf. |
| 36 | ((radiotherap* or radio-therap* or (radiation adj3 therap*) or chemoradi* or radiochemo* or chemo-radi* or radio-chemo* or irradiat* or brachytherapy) adj5 (adverse* or toxic* or "side effect*" or safety or injur* or induced)).ab. |
| 37 | ((acute or late) adj4 (reaction* or toxicit*)).ab. and (radiotherap* or radio-therap* or (radiation adj3 therap*) or chemoradi* or radiochemo* or chemo-radi* or radio-chemo* or irradiat* or brachytherapy).ab. /freq=3 |
| 38 | CTCAE.ti,ab,kw. |
| 39 | ("common terminology criteria" adj3 "adverse events").ti,ab,kw. |
| 40 | or/20-39 [treatment outcome: survival recurrence toxicity] |
| 41 | 19 and 40 [Cervical cancer + radiation + outcomes] |
| 42 | exp lymphocytes/ |
| 43 | lymphocyte*.ti,kf. |
| 44 | (lymphocyte* adj3 (count* or nadir or ratio or ratios)).ti,ab,kf. |
| 45 | exp Lymphocyte Activation/ |
| 46 | exp Lymphocyte Count/ |
| 47 | exp platelet count/ |
| 48 | exp Leukocytes/ |
| 49 | Leukocyte count/ |
| 50 | exp Neutrophils/ |
| 51 | (Leukocytes or "leukocyte count*").ti,ab,kf. |
| 52 | ((platelet* or neutrophil* or granulocyte* or eosinophil* or monocyte*) adj5 lymphocyte*).ti,ab,kf. |
| 53 | Neutrophil*.ti,ab,kf. |
| 54 | monocyte*.ti,ab,kf. |
| 55 | (monocyte:lymphocyte or lymphocyte:monocyte).ti,ab,kf. |
| 56 | "platelet count*".ti,ab,kf. |
| 57 | platelet:lymphocyte.ti,ab,kf. |
| 58 | platelet*.ab. /freq=3 |
| 59 | (hematologic* adj2 (biomarker* or parameter*)).ti,kf. |
| 60 | (immunity or immune or immunological).ti. |
| 61 | exp Lymphopenia/ |
| 62 | (lymphocytopenia* or lymphopenia*).ti,ab. |
| 63 | or/42-62 [ immunological status; lymphocytes;] |
| 64 | 41 and 63 [Cervical cancer radiation therapy + treatment outcomes + lymphocyte count, lymphocyte ratio etc.] |
| 65 | exp antigens, differentiation, t-lymphocyte/ |
| 66 | exp T-Lymphocytes/ |
| 67 | (T-Lymphocyte* or "t-cell*").ti,ab,kf. |
| 68 | (CD4* or CD8* or CD3*).ti,kf. |
| 69 | exp Cytotoxicity, Immunologic/ |
| 70 | (cytotoxicity adj3 immunologic).ti,ab,kf. |
| 71 | exp b7 antigens/ |
| 72 | exp Immunity, Cellular/ |
| 73 | ((cell or cellular) adj3 (immunity or immunologic)).ti,ab,kf. |
| 74 | exp Biomarkers/im |
| 75 | or/65-74 |
| 76 | 19 and 75 [Cervical cancer radiation therapy + immune markers] |
| 77 | exp HIV/ |
| 78 | exp HIV Infections/ |
| 79 | (human adj3 ("immuno deficiency" or immunodeficiency) adj3 virus*).ti,ab,kf. |
| 80 | (hiv or aids).ti,ab,kf. |
| 81 | (acquired adj3 ("immune deficiency" or immunodeficiency) adj3 syndrome*).ti,ab,kf. |
| 82 | or/77-81 [HIV positive] |
| 83 | 19 and 82 [Cervical cancer radiation therapy + HIV] |

**Appendix 2. Summary and full grading per** NIH National Heart, Lung, and Blood Institute quality assessment tool for observational cohort studies

| Study | Q1 | Q2 | Q3 | Q4 | Q5 | Q6 | Q7 | Q8 | Q9 | Q10 | Q11 | Q12 | Q13 | Q14 | P/R |
| --- | --- | --- | --- | --- | --- | --- | --- | --- | --- | --- | --- | --- | --- | --- | --- |
| Table 1 | | | | | | | | | | | | | | | |
| Cho et al, 2016 | Y | Y | Y | N | N | Y | Y | Y | Y | Y | Y | N | N | Y | R |
| Mizunuma et al, 2015 | Y | Y | Y | Y | N | Y | Y | N | Y | N | Y | N | N | Y | R |
| Jonka-Gmyrek et al, 2018 | Y | Y | Y | N | N | Y | Y | N | Y | Y | Y | N | N | Y | R |
| Holub & Biete, 2019 | Y | Y | Y | Y | N | Y | Y | N | Y | N | Y | N | N | Y | R |
| Glicksman et al, 2017 | Y | Y | Y | N | N | Y | Y | Y | Y | Y | Y | N | N | Y | P |
| Choi et al, 2008 | Y | Y | Y | Y | N | Y | N | Y | Y | N | Y | N | N | Y | R |
| Hoskin et al, 2014 | Y | Y | Y | Y | N | Y | Y | N | Y | Y | Y | N | N | Y | R |
| Haraga et al, 2016 | Y | Y | Y | Y | N | Y | Y | Y | Y | N | Y | N | N | Y | R |
| Koulis et al, 2017 | Y | Y | Y | Y | N | Y | Y | N | Y | N | Y | N | N | Y | R |
| Lee et al, 2020 | Y | Y | Y | N | N | Y | Y | N | Y | N | Y | N | N | Y | R |
| Wisdom et al, 2019 | Y | Y | N | Y | N | Y | Y | Y | Y | Y | Y | N | N | Y | R |
| Taguchi et al, 2020 | Y | Y | Y | Y | N | Y | Y | N | Y | Y | Y | N | N | Y | R |
| Petrillo et al, 2015 | Y | Y | Y | Y | N | Y | N | Y | Y | Y | Y | N | N | Y | P |
| Wu et al, 2016 | Y | Y | Y | Y | Y | Y | N | Y | Y | Y | Y | N | N | Y | R |
| Onal et al, 2016 | Y | Y | Y | Y | Y | Y | N | N | Y | Y | Y | N | N | Y | R |
| Onal et al, 2018 | Y | Y | Y | Y | N | Y | Y | N | Y | Y | Y | N | N | Y | R |
| Escande et al, 2016 | Y | Y | Y | Y | N | Y | Y | N | Y | Y | Y | N | N | Y | P |
| Wang et al, 2016 | Y | Y | Y | Y | N | Y | Y | N | Y | Y | Y | N | N | Y | R |
| Li et al, 2016 | Y | Y | Y | N | N | Y | Y | N | Y | Y | Y | N | N | Y | R |
| Cho et al, 2017 | Y | Y | Y | N | N | Y | Y | N | Y | N | Y | N | N | Y | R |
| Lee et al, 2019 | Y | Y | N | Y | N | Y | Y | N | Y | N | Y | N | N | Y | R |
| Jeong et al, 2019 | Y | Y | Y | N | N | Y | Y | N | Y | N | Y | N | N | Y | R |
| Table 2 | | | | | | | | | | | | | | | |
| Ordoñez et al, 2013 | Y | Y | Y | N | N | Y | Y | N | Y | N | Y | N | N | Y | P |
| Yang et al, 2006 | Y | Y | Y | N | N | Y | CD | N | Y | Y | Y | N | N | Y | P |
| Delgado et al, 2009 | Y | Y | Y | N | N | Y | N | N | Y | Y | Y | N | N | N | P |
| Ma et al, 2018 | Y | Y | Y | N | N | Y | Y | N | Y | N | Y | N | N | Y | P |
| Tsuchiya et al, 2019 | Y | Y | Y | Y | N | Y | Y | N | Y | Y | Y | Y | N | Y | R |
| Matsumoto et al, 2017 | Y | Y | Y | Y | N | Y | Y | Y | Y | N | Y | Y | N | Y | R |
| Cosper et al, 2020 | Y | Y | N | N | N | Y | Y | Y | Y | Y | Y | Y | N | N | P |
| Petrillo et al, 2015 | Y | Y | Y | Y | N | Y | N | N | Y | N | Y | N | N | Y | P |
| Martins et al, 2019 | Y | Y | Y | N | N | Y | Y | Y | Y | N | Y | Y | N | N | R |
| Table 3 | | | | | | | | | | | | | | | |
| Siraprapasiri et al, 2011 | Y | Y | Y | N | N | Y | Y | N | Y | NA | Y | N | Y | N | P |
| Grover et al, 2018 | Y | Y | Y | Y | Y | Y | Y | Y | Y | NA | Y | N | Y | Y | P |
| Vendrell et al, 2018 | Y | Y | Y | Y | N | Y | Y | Y | Y | NA | Y | N | Y | Y | R |
| Simonds et al, 2015 | Y | Y | Y | N | N | Y | Y | Y | Y | NA | Y | N | Y | Y | P |
| Einstein et al, 2019 | Y | Y | Y | Y | Y | Y | Y | N | Y | NA | Y | N | Y | Y | P |

*Abbreviations*: CD, couldn’t determine; N, No; NA, not applicable; P, prospective; R, retrospective Y, yes

Questions:

1. Was the research question or objective in this paper clearly stated?

2. Was the study population clearly specified and defined?

3. Was the participation rate of eligible persons at least 50%?

4. Were all the subjects selected or recruited from the same or similar populations (including the same time period)? Were inclusion and exclusion criteria for being in the study prespecified and applied uniformly to all participants?

5. Was a sample size justification, power description, or variance and effect estimates provided?

6. For the analyses in this paper, were the exposure(s) of interest measured prior to the outcome(s) being measured?

7. Was the timeframe sufficient so that one could reasonably expect to see an association between exposure and outcome if it existed?

8. For exposures that can vary in amount or level, did the study examine different levels of the exposure as related to the outcome (e.g., categories of exposure, or exposure measured as continuous variable)?

9. Were the exposure measures (independent variables) clearly defined, valid, reliable, and implemented consistently across all study participants?

10. Was the exposure(s) assessed more than once over time?

11. Were the outcome measures (dependent variables) clearly defined, valid, reliable, and implemented consistently across all study participants?

12. Were the outcome assessors blinded to the exposure status of participants?

13. Was loss to follow-up after baseline 20% or less?

14. Were key potential confounding variables measured and adjusted statistically for their impact on the relationship between exposure(s) and outcome(s)?
